# Effects of a reduction of the number of electrodes in the EEG montage on the number of identified seizure patterns

**DOI:** 10.1038/s41598-022-08628-9

**Published:** 2022-03-17

**Authors:** Moritz Tacke, Katharina Janson, Katharina Vill, Florian Heinen, Lucia Gerstl, Karl Reiter, Ingo Borggraefe

**Affiliations:** 1 Division of Pediatric Neurology, Developmental Neurology and Social Pediatrics, Department of Pediatrics, Dr. von Hauner Children’s Hospital, University Hospital LMU Munich, Lindwurmstr. 4, 80337 Munich, Germany; 2 Epilepsy Center, University Hospital LMU Munich, Munich, Germany; 3 Division of Pediatric Intensive Care, Department of Pediatrics, Dr. von Hauner Children’s Hospital, University Hospital LMU Munich, Lindwurmstr. 4, 80337 Munich, Germany

**Keywords:** Neurological disorders, Paediatric research

## Abstract

Continuous EEG monitoring (cEEG) is frequently used in neurocritical care. The detection of seizures is one of the main objectives. The placement of the EEG electrodes is time consuming, therefore a reduced montage might lead to an increased availability in the ICU setting. It is unknown whether such a reduction of electrodes reduces the number of seizure patterns that are detected. A total of 95 seizure and 95 control EEG sequences from a pediatric epilepsy monitoring unit (EMU) were anonymized and reduced to an eight-lead montage. Two experts evaluated the recordings and the seizure detection rates using the reduced and the full montage were compared. Sensitivity and specificity for the seizure detection were calculated using the original EMU findings as gold standard. The sensitivity to detect seizures was 0.65 for the reduced montage compared to 0.76 for the full montage (p = 0.031). The specificities (0.97 and 0.96) were comparable (p = 1). A total of 4/9 (44%) of the generalized, 12/44 (27%) of the frontal, 6/14 (43%) of the central, 0/1 (0%) of the occipital, 6/20 (30%) of the temporal, and 5/7 (71%) of the parietal seizure patterns were not detected using the reduced montage. The median time difference between the onset of the seizure pattern in the full and reduced montage was 0.026s (IQR 5.651s). In this study the reduction of the EEG montage from 21 to eight electrodes reduced the sensitivity to detect seizure patterns from 0.76 to 0.65. The specificity remained virtually unchanged.

## Introduction

About a fourth of the admissions in a general pediatric intensive care unit (PICU) is due to neurological diseases and symptoms^1^. Continuous neurological monitoring is a cornerstone in the management of these patients^2,3^. This includes continuous EEG (cEEG) monitoring, mainly to assess the state of consciousness/encephalopathy and to identify seizures^4,5^. Seizures can be a sign for a potentially treatable deterioration of critical ill children (e.g. an intracranial hemorrhage). A symptomatic treatment of seizures is recommended, or at least seen as appropriate, in many situations, e.g. traumatic brain injuries and the post-cardiac-arrest-syndrome^6,7^. Prompt reactions to the occurrence of seizures require a timely diagnosis, and the high number of non-convulsive seizures as well as the use of neuromuscular blockade can inhibit the reliable clinical identification of seizures on the PICU.

In such situations the cEEG can be the only way to detect seizures. However, the use of the cEEG leads to an increased workload on the ICU staff. Especially the placement of the electrodes is a time-consuming task. Therefore, the use of a reduced EEG montage, with a lower number of electrodes, saves time and increases availability. A lower number of electrodes leads to the recording of less data, which might compromise the diagnostic value of the method.

Generalized phenomena, e.g. the patterns showing the grade of encephalopathy, can be recognized using two or four EEG electrodes (as shown by the established use of the aEEG, a processed two- or four-lead-EEG, in the context of hypoxic-ischemic encephalopathy after neonatal asphyxia^8^). Localized processes like focal seizure patterns might not be captured by montages with a reduced set of electrodes. The estimations on the number of missed seizures in montages with a reduced electrode set vary: in a study with very limited EEG durations (15s), the ability of neurological experts to detect seizure patterns was not reduced in a montage consisting of eight electrodes compared to a full montage^9^. In contrary, data on neonates showed that a reduction in the electrode count leads to a significant decrease in the number of seizures that were detected^10^. A recent study on critically ill adults showed that the use of a reduced, circular ten-lead montage lead to a sensitivity of 67% for the detection of seizures^11^. A small series on adults suggested the possible use of a six-lead montage as a screening tool for non-convulsive status epilepticus in the emergency department^12^.

**Figure 1:**
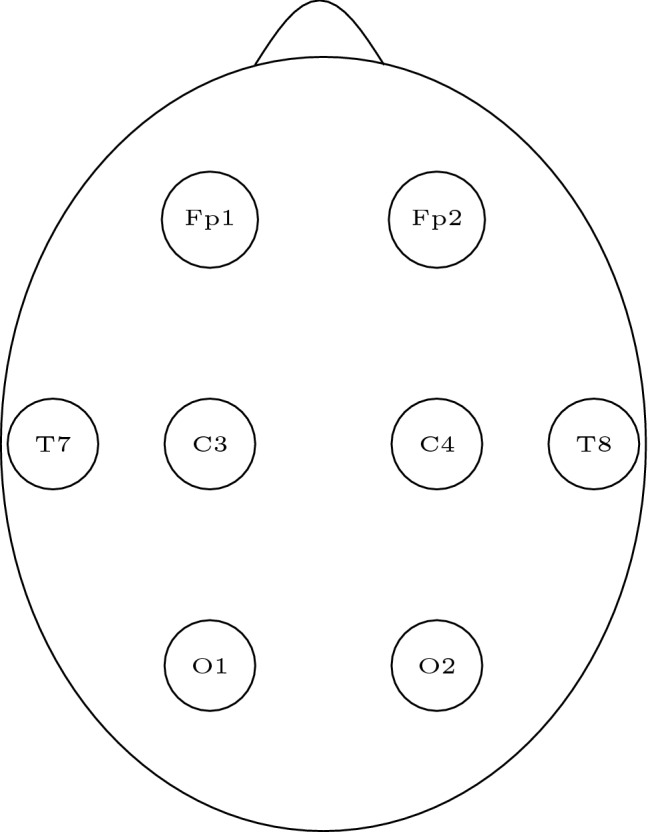
The electrodes used in the reduced montage.

The study presented here uses an epilepsy monitoring unit (EMU) derived data set to quantify the influence of a reduced montage with only eight electrodes on the seizure detection rate in a pediatric population. This montage uses a frontal, a central, a temporal and an occipital electrode on each side, see Fig. 1. It is in accordance with the German guidelines to diagnose brain death^13^ and is therefore an established montage in the ICU setting.

The advantage of the use of EMU-derived data is that the EMU employs a maximum of resources to detect seizures. This includes a continuous EEG and video monitoring by a dedicated staff as well as the cooperation of the patients and their caregivers, reporting all events including those that might be missed on the video (e.g. purely subjective seizures). Therefore, the seizure detection rates on the EMUs can be assumed to be at the highest attainable level.

## Methods

This study was approved by the Munich university’s institutional review board under the number 18-404 on February 18th, 2019. Informed consent for retrospective EEG analysis was obtained from the parents or legal guardian(s) of the children. All the relevant regulations from the declaration of Helsinki were applied, as were all applicable data protection regulations, including a full anonymization.

The source data set were video EEG recordings from all children younger than 18 years that were admitted to the epilepsy monitoring unit (EMU) between 2004 and 2018 where consent was given. The reason for the EMU evaluations were most frequently medication-refractory seizures or paroxysmal events of unknown origin. In the course of the stay on the EMU the EEG was continuously monitored. All paroxysmal events along with their EEG classifications were documented. Due to disk space limitations, only parts of the continuous EEG recordings (usually covering several days for every child) were permanently stored in the EEG archives. All stored EEG recordings containing seizure patterns were identified. From these, 95 seizures were randomly drawn. For every seizure, two EEG sequences, each with a duration of 30 minutes, were selected. One sequence covered the seizure pattern, the other sequence was a seizure-free control sequence. The control sequences were ideally recorded exactly 24h before the seizure. If this sequence was not present, or not usable, another control sequence was selected that was recorded at a similar time of day as the seizure. This data set has previously been described^14^. For details, refer to this publication.

**Table 1:** Overview of the EEG files present for a single seizure along with the assignment to the two reviewers.

	EEG montage	
	Full	Reduced
Seizure	Reviewer A	Reviewer B
Control	Reviewer B	Reviewer A

In this case, Reviewer A was selected to review the full montage EEG containing the seizure pattern.

Every EEG sequence was converted to the European Data Format (EDF). The data were digitally resampled to a reduced montage (FP1/2, C3/4, T7/8, O1/2, reference, and ECG leads, see Fig. 1). Therefore, each EEG sequence existed with the reduced montage as well as with the full montage (FP1/2, F3/4/7/8, C3/4, T7/8, P3/4/7/8, O1/2, TP9/10, Fz, Cz, Pz, reference, and ECG leads). Two reviewers (IB, MT), both board-certified in EEG evaluation, board-certified epileptologists, and with expertise in longtime video-EEG monitoring reviewed the EEGs without any access to further information (e.g. videos, EEG annotations, patient details). Each full-montage EEG containing a seizure was randomly assigned to one reviewer, the other sequences for this seizure were then systematically distributed to the reviewers, see Table 1.

The “EDFbrowser” version 1.67 (https://www.teuniz.net/edfbrowser/), a free EEG evaluation software, was used to review the EEGs. The reviewers annotated the files regarding the presence of epileptic seizure patterns. If a pattern was identified, an annotation was set to the beginning of the seizure pattern. Lateralization and localization of the seizure pattern start were noted. After reviewing, all EEG files were scanned using a custom software. The annotations were extracted, and analyzed.

For the following statistical evaluations, the findings from the EMU reports were used as the gold standard against which the interpretations of both the reduced and the full montage were compared. The main endpoint analyzed was the detection rate of the seizure patterns. Sensitivity and specificity of both montages were compared using the McNemar test. For each localization, the proportion of missed seizures was calculated. Fisher’s exact test was used to examine these proportions.

The posterior probability distributions for the sensitivity and specificity were modeled in a Bayesian fashion. Modeling the detection of the seizure patterns as a binomial process, the beta distribution was used as conjugate prior^15^. A “flat” prior, i.e. using the parameter values $a=1$ and $b=1$, was employed. The resulting posterior distributions were plotted.

In an exploratory analysis, the concordance of the localization (given as “frontal”, “temporal”, “central”, “parietal”, “occipital”, or “generalized”) and the lateralizations (“right”, “left”, “not lateralized”) were compared using the McNemar test. A Bonferroni correction was employed, therefore all p values less than 0.0125 were considered to be statistically significant. “R” version 4.0.4 was used for the calculations.

## Results

The data set consisted of 95 pairs of seizure and control sequences from 36 patients, 20 of them male, median age 11.9 years. Using the reduced EEG montage, the sensitivity to detect a seizure was 0.653 and the specificity was 0.968. The results of the full montage were 0.758 and 0.958 respectively (see^14^). The p-values calculated using the McNemar-Test to compare the sensitivities and specificities were 0.031 and 1, respectively. These differences were not statistically significant (p-values 0.031 and 1, respectively, not statistically significant due to a bonferroni correction to a p-level of 0.0125). The contingency tables for the correct assessment as seizure and control EEGs are displayed in the Tables 2a and b, respectively.

**Figure 2:**
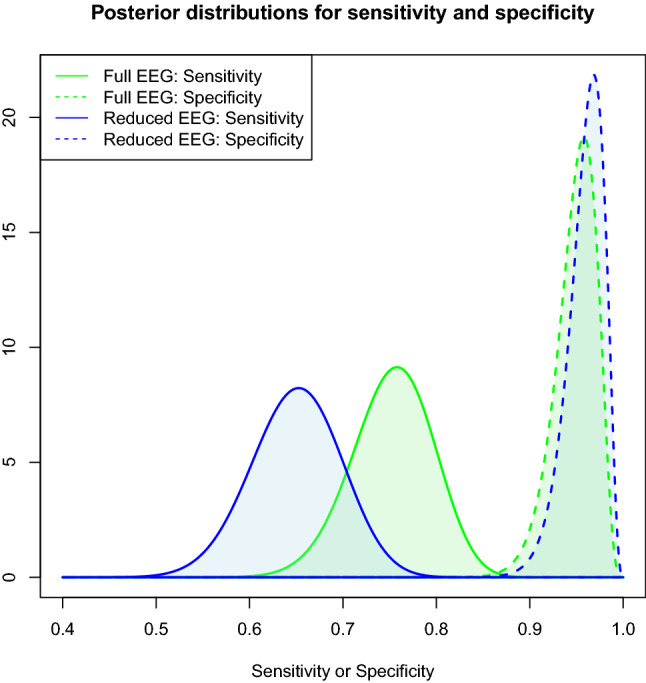
Posterior distributions for the sensitivity and the specificity of both EEG montages. Similar to the use of confidence intervals this plot shows the estimations for the sensitivities and specificities. The lower, and wider, curves for the sensitivities signify that, given the observed data (i.e. the EEG classifications), there is still a relevant degree of uncertainty regarding the true sensitivities.

Figure 2 shows the posterior probability distributions for the sensitivity and specificity using both montages.

Using the localizations and lateralizations from the original EMU evaluations, 4/9 (44%) of the generalized, 12/44 (27%) of the frontal, 6/14 (43%) of the central, 0/1 (0%) of the occipital, 6/20 (30%) of the temporal, and 5/7 (71%) of the parietal seizure patterns were not identified using the reduced montage. Comparing these rates using Fisher’s exact test lead to a p-value of 0.23).

**Table 2:** The contingency tables comparing the correctness rates on the seizure and control EEGs.

	Reduced montage		
	Correct	Incorrect	
**(a) Correct classification of an EEG as a “seizure” EEG, p-value: 0.031**			
Full montage correct	58	14	72
Full montage incorrect	4	19	23
	62	33	95
**(b) Correct classification of an EEG as a “control” EEG, p-value: 1**			
Full montage correct	88	3	91
Full montage incorrect	4	0	4
	92	3	95

The correctness of localization and lateralization was assessed for all identified seizure patterns. Table 3a (for the localizations) and b (lateralizations) show how frequent the seizure onset zones that were diagnosed using the full and reduced montages were identical to the results from the original EMU evaluations. The localizations assessed using the full and reduced montage were consistent with the original assessments in 40% and 26% of the seizures, the lateralizations in 50% and 45%, respectively.

**Table 3:** The contingency tables show the frequencies of correct localization and lateralization compared to the original EMU classifications.

	Reduced montage		
	Correct	Incorrect	
**(a) Localization correctness, p-value: 0.189**			
Full montage correct	9	14	23
Full montage incorrect	7	28	35
	16	42	58
**(b) Lateralization correctness, p-value: 0.581**			
Full montage correct	21	8	29
Full montage incorrect	5	24	29
	26	32	58

**Figure 3:**
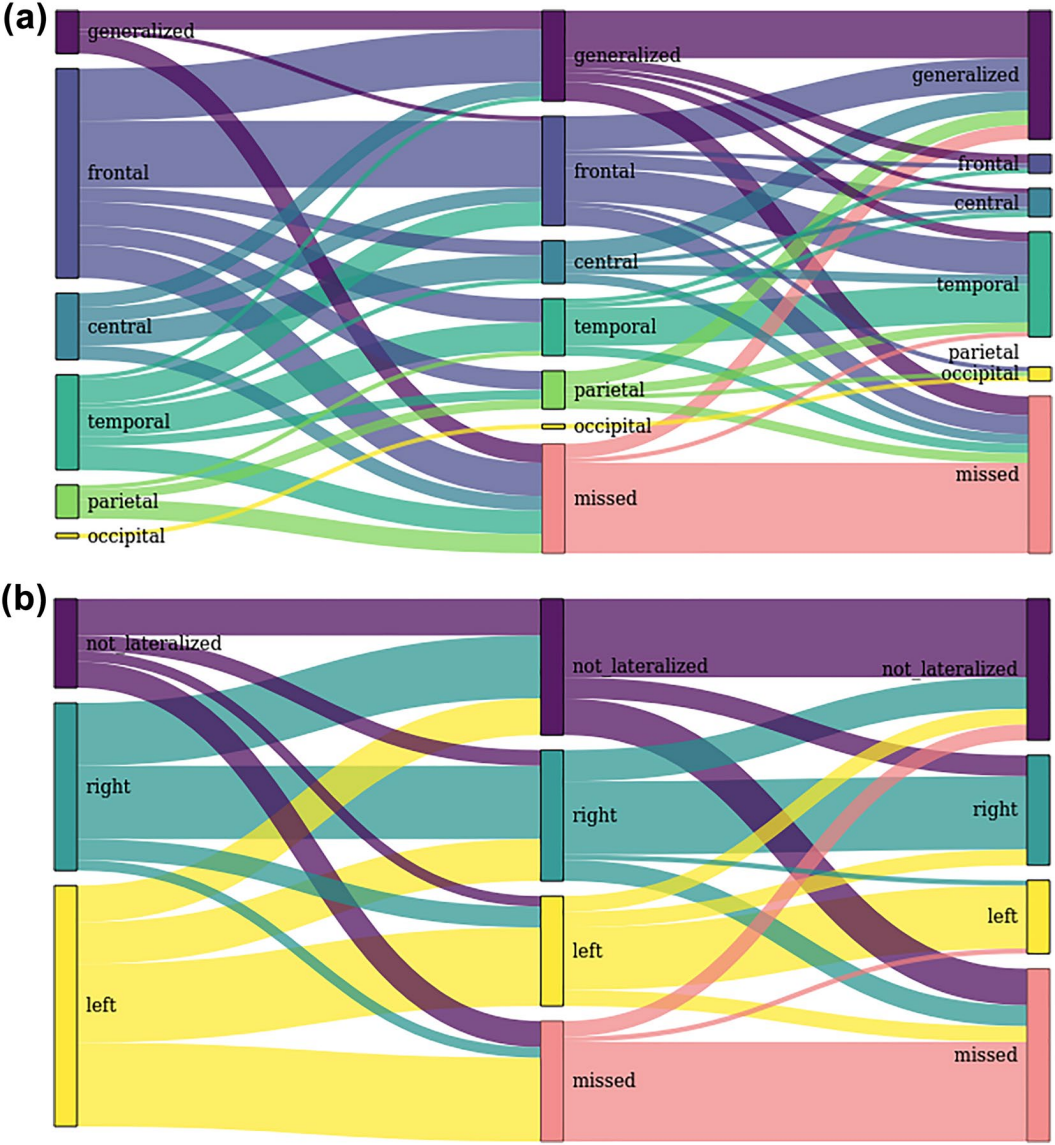
Sankey diagram depicting differences between the localizations and lateralizations of the epileptic seizure patterns between the original EMU classifications (left), the assessment of the full EEG montage (middle), and the reduced EEG montage (right).

Figure 3 shows how the diagnosed localization and lateralization of the seizure patterns changed depending on the montage used. Most seizure patterns that were not identified—compared to the EMU evaluations—using the full montage could neither be detected with the reduced montage. Regarding the seizure patterns that were detected using the full montage, but missed with the reduced montage, no clear pattern emerges as all localizations and lateralizations were affected. The figure further illustrates a high degree of variability regarding the identification of the seizure onset zone. The number of seizure pattern that were classified as “generalized” increased: in the EMU evaluations, 9 generalized seizure patterns were detected, compared to 19 using the full and 27 with the reduced montage.

The median time difference between the seizure pattern onset as diagnosed in the reduced and full montage was 0.026 s (IQR: 5.651 s), the time difference exceeded 30 s in 2 cases (3.4%).

## Discussion

The results of this study suggest that reducing the EEG montage from 21 electrodes to eight electrodes leads to a lower number of detected seizure patterns. Sensitivity fell from 0.758 to 0.653. Specificity was virtually unchanged at 0.968 (full montage: 0.958). When examining the influence of the seizure pattern’s localization on the rate of missed seizures, a minimum of 0/1 (0%) occipital seizure patterns and a maximum of 5/7 (71%) parietal seizure patterns were missed. It is noteworthy that these were the two localizations with the lowest number of seizures. For all other localizations, the rate of missed seizure patterns was between 27% and 44%. Therefore, using this data, no reliable identification of localizations where more or less seizure patterns are missed using the reduced montage could be made.

The comparison of the localization and lateralization of the detected seizure patterns between the full and the reduced EEG montage showed no significant differences. The number of seizures that were classified as “generalized” increased with every step of data reduction. It went up from 9 instances in the EMU assessment to 19 using the full montage (but, contrary to the EMU, without access to additional data like video recordings). On the reduced montage, 27 seizure patterns were noted as being generalized. This is compatible with the occurrence of secondary generalization: the focal onset of the seizure pattern is missed using the reduced montage while the later, generalized period of the seizure pattern is detected. The diagnosis of the lateralization and localization differed from the original assessment in the EMU in at least half of the cases. This shows the high degree of inter-rater variability that is inherent to EEG evaluations and is illustrated by Fig. 3.

There was no relevant time difference between the markings set to the start of the seizure pattern in the reduced and full montage EEGs. This can be seen as a indirect hint that there is no tendency to overlook short seizure patterns in the reduced montage. In two seizures the time difference exceeded 30 s. This suggests that, in one of the two montages, the “real” seizure pattern was missed and additionally a false positive occurred. Conversely the fact that there were only two such events also means that in all other cases the same seizure pattern was recognized in the two different montages.

The main limitation of this study derives from the fact that it attempts to answer a PICU-related question using non-PICU-data. There are differences in the EEGs recorded on the PICU and in the epilepsy monitoring unit. In PICUs, patients with seizures will frequently also have an acute encephalopathy and show the respective changes in the EEGs. On the epilepsy monitoring unit, acute encephalopathy is rare and mainly seen in post-ictal patients. However, there are justifications for the approach presented here. By its very nature, in the PICU it is difficult to create a data set of comparable quality. On the PICU, there is no dedicated staff that continuously monitors both the patient and the EEG for seizures. While individual seizure semiology in EMU patients is known to parents and professionals, acutely ill children on the ICU typically have the first seizures of their lives^16,17^, which might be missed clinically. Non-convulsive seizures and seizures in the presence of a neuromuscular blockade might be entirely missed. As the presence of clinical information about the seizures influences the number of seizure patterns that are recognized as such^14^, the lower number of clinically recognizable seizures might lead to a reduced seizure detection rate in the PICU setting. Studies quantifying the prevalence of seizures on the PICU^18^ might report a lower bound on the real seizure burden.

The results presented here imply that a fraction of the seizure patterns in the range of 10% may be missed when using an eight-lead montage compared to a full montage. This seems like a sensible trade-off given that the number of electrodes could be reduced by more than half, significantly lowering the time needed to start and run the cEEG monitoring. It should be noted that a reduced electrode set does not help in the evaluation of the EEG recordings. This issue can be addressed in multiple ways: The use of the so-called “quantitative EEG”, i.e. the use of computerized transformations on the data to aid the interpretation of the EEG^5,19^, or the application of automated seizure detection systems. The latter have, to our knowledge, not yet been evaluated in the context of pediatric ICU EEG monitoring.

## Conclusions

Taken together, a reduced EEG montage with eight leads might be a viable compromise that balances the time needed to place the electrodes and the suitability of the resulting recordings to detect seizure patterns.
